# Oxidized Lipids: Common Immunogenic Drivers of Non-Alcoholic Fatty Liver Disease and Atherosclerosis

**DOI:** 10.3389/fcvm.2021.824481

**Published:** 2022-01-10

**Authors:** Constanze Hoebinger, Dragana Rajcic, Tim Hendrikx

**Affiliations:** ^1^Department of Laboratory Medicine, Klinisches Institut für Labormedizin (KILM), Medical University Vienna, Vienna, Austria; ^2^Department of Molecular Genetics, School of Nutrition and Translational Research in Metabolism (NUTRIM), Maastricht University, Maastricht, Netherlands

**Keywords:** NAFLD (non-alcoholic fatty liver disease), oxidized lipids, foamy macrophages, immunoglobulins, atherosclerosis, NASH (non-alcoholic steatohepatitis)

## Abstract

The prevalence of non-alcoholic fatty liver disease (NAFLD), ranging from simple steatosis to inflammatory steatohepatitis (NASH) and cirrhosis, continues to rise, making it one of the major chronic liver diseases and indications for liver transplantation worldwide. The pathological processes underlying NAFLD not only affect the liver but are also likely to have systemic effects. In fact, growing evidence indicates that patients with NAFLD are at increased risk for developing atherosclerosis. Indeed, cardiovascular complications are the leading cause of mortality in NAFLD patients. Here, we aim to address common pathophysiological molecular pathways involved in chronic fatty liver disease and atherosclerosis. In particular, we focus on the role of oxidized lipids and the formation of oxidation-specific epitopes, which are important targets of host immunity. Acting as metabolic danger signals, they drive pro-inflammatory processes and thus contribute to disease progression. Finally, we summarize encouraging studies indicating that oxidized lipids are promising immunological targets to improve intervention strategies for NAFLD and potentially limit the risk of developing atherosclerosis.

## Introduction

A sedentary lifestyle and excess caloric intake combined with reduced energy expenditure not only lead to overweight and obesity but also an increase in the prevalence of metabolic syndrome and various lipid-mediated diseases such as non-alcoholic fatty liver disease (NAFLD) (1, 2). As the liver is the most metabolic organ in the human body, increased circulatory lipid levels result in their accumulation in the liver, known as steatosis (fat accumulation in >5% of hepatocytes) (3, 4). While simple steatosis is still reversible by lifestyle modifications, the NAFLD spectrum also includes more progressive non-alcoholic steatohepatitis (NASH), characterized by inflammation, hepatocyte damage, and fibrosis (5). Importantly, the presence of NASH further increases the risk of developing an end-stage liver disease such as cirrhosis or hepatocellular carcinoma, ultimately requiring liver transplantation (4). By affecting ~25% of individuals, both adults and children, NAFLD has become the leading cause of chronic liver disease worldwide (6, 7). Considering that the prevalence is expected to rise further (7), NAFLD can be regarded as significant health and economic burden worldwide, resulting in a reduced quality of life (8).

While NAFLD primarily affects liver structure and function, leading to morbidity and mortality from liver failure, cardiovascular disease (CVD) is the most common cause of death in early NAFLD patients (9, 10). Moreover, there is increasing evidence that NAFLD is a risk factor for developing cardiovascular complications such as atherosclerosis (11). Atherosclerosis can be broadly described as a progressive chronic inflammatory disease of the large and medium-sized arteries that share metabolic patterns with NAFLD (12, 13). More precisely, atherosclerosis is characterized by the thickening and hardening of the arterial walls, mainly caused by developing complex lesions and accumulation of lipids and fibrous elements known as atheromatous plaques narrowing the arterial lumen (14, 15). Subsequently, plaque rupture and thrombosis can lead to acute clinical complications, such as heart attacks, strokes, unstable angina, arrhythmia, or sudden cardiac death, making CVD the primary cause of morbidity and mortality in Western countries (16).

Since atherosclerosis and NAFLD co-occur in patients with the metabolic syndrome, obesity, type 2 diabetes mellitus and insulin resistance, it is difficult to decipher the exact cause-effect relationship that leads to an increased risk of CVD in patients with NAFLD (17). Recent studies in children suffering from fatty liver disease support that NAFLD may cause CVD (18–20). Nevertheless, while growing evidence indicates that NAFLD can be considered a risk factor for atherosclerosis, the underlying disease mechanisms by which NAFLD contributes to CVD are not entirely understood. In this review, we will focus on the immunomodulatory effects of oxidized lipids and provide evidence for their involvement as common metabolic triggers for disease progression during NAFLD and atherosclerosis. In addition, we will discuss how targeting oxidized lipids *via* immunization strategies can be explored to improve interventions and potentially prevent the risk for CVD.

## Dyslipidemia and the Formation of Oxidation-Specific Epitopes

The defining hallmark of NAFLD is the accumulation of lipids containing triglycerides, cholesterol esters, and other lipid species in the liver. The increased hepatic triglyceride content, which determines the histological appearance of a steatotic liver, is a consequence of increased calorie intake, enhanced free fatty acids (FFA) influx from lipolysis of peripheral adipose tissue, elevated triglyceride synthesis by hepatic *de novo* lipogenesis, and reduced lipid export from the liver *via* very-low-density lipoprotein (VLDL) particles (21, 22). In parallel with deregulated hepatic lipid metabolism, NAFLD is associated with systemic dyslipidemia, as manifested by elevated triglyceride and cholesterol levels, lowered high-density lipoproteins (HDL), and increased low-density lipoprotein (LDL) particles in circulation (23–25). Similarly, elevated cholesterol, high LDL, and low HDL serum levels are described as risk factors for the onset of CVD (26), where the accumulation of LDL particles in arterial walls is a crucial process in the development of atherosclerosis (27). Thus, despite the strong correlation with dyslipidemia, it has become clear that disturbances in lipid metabolism and increased LDL levels cannot merely explain the local pro-inflammatory tissue environment, of which its presence seems to be a crucial factor enhancing disease progression.

Dyslipidemia that goes beyond the body's coping mechanisms can lead to lipotoxicity, an essential mechanism associated with NAFLD and atherosclerosis (28, 29). During NAFLD, lipotoxicity occurs when the massive influx of FFAs into hepatocytes peaks at a point where the liver can no longer use or store the FFAs or export them as triglycerides. Subsequently, a chain of intracellular responses is activated, leading to lipotoxic stress in mitochondria and the endoplasmic reticulum, ultimately resulting in hepatocyte cell death and the release of pro-inflammatory cytokines and extracellular vesicles (28, 30–33). In turn, this leads to the activation of resident Kupffer cells and the recruitment of infiltrating monocytes and neutrophils to the liver, which contribute to inflammation *via* the release of cytokines, chemokines, nitric oxide, and reactive oxygen species (ROS) (4). In a similar process, lipid retention in atherosclerotic plaques induces local inflammation characterized by the influx of circulating monocytes that differentiate into macrophages that release pro-inflammatory stimuli and ROS (4, 34). Although ROS are products of normal cell metabolism and serve as signal molecules as in redox signal pathways (35), continued oxidative stress, characterized by high ROS exposure in combination with reduced levels or scavenging capacity of antioxidants, will harm different vital macromolecules such as proteins, nucleic acids (DNA/ RNA), and lipids (36).

Especially phospholipids, as building blocks of cells, and lipoproteins, are popular targets of ROS as part of a process called lipid peroxidation, which occurs *via* both enzymatic and non-enzymatic mechanisms (37, 38). Whereas, the enzymatic process of lipid peroxidation covers the activation of myeloperoxidases, lipoxygenases, cyclooxygenases, and cytochrome p450 (38, 39), the non-enzymatic process requires free radicals. Therefore, it can only be activated indirectly *via* nicotinamide adenine dinucleotide phosphate (NADPH) oxidases and nitric oxide synthases (40). Both processes result in lipid hydroperoxide molecules, which are then degraded. Notably, a large variety of secondary products are formed during the degradation process of lipid peroxidation, including malondialdehyde (MDA), malondialdehyde-acetaldehyde (MAA), 4-hydroxynonenal (4-HNE), and the remaining core aldehyde of oxidized phospholipids (OxPL) (41–43). These oxidized lipids and their degradation products can hamper the normal function of proteins and lipids and therefore modify them (44). Further, some of these lipid derivatives, such as highly reactive aldehydes, can alter their self-molecules and form so-called oxidation-specific epitopes (OSEs), which comprise protein adducts with degradation products of lipid peroxidation, such as MDA and phosphocholine-containing OxPL (PC-OxPL) (34, 45). If removal of these products, primarily carried by dying cells, extracellular vesicles, and damaged lipoproteins such as oxidized LDL (OxLDL), is insufficient, sterile inflammation is triggered, and oxidative damage is exacerbated (37, 46, 47).

Mounting evidence indicates increased levels of oxidized lipids and elevated presence of various OSEs during the progression from simple steatosis to NASH (46, 48–50) as well as in atherosclerosis and CVD (47). Of the different types of OSEs that can be formed during lipid peroxidation, MDA and 4-HNE are prototypical markers of oxidative stress that can be measured, for example, by the commonly used 2-thiobarbituric acid reaction (TBAR) assay (37). While the presence of 4-HNE is associated with different stages of fatty liver disease (51) and mitochondrial 4-HNE adducts are increased in NASH (52), 4-HNE is also found in atherosclerotic lesions in humans and animal models of disease (34). Furthermore, MDA epitopes are increased in patients, mice, and rats suffering from NAFLD and NASH (53–58). In addition to elevated systemic MDA concentrations, we previously demonstrated that MDA adducts accumulate in the liver during human NASH and in hypercholesterolemic *Ldlr*^−/−^ mice with steatohepatitis (46, 59). Similarly, atherosclerotic lesions were shown to contain MDA epitopes (46), and elevated serum MDA-LDL levels are associated with the progression of carotid atherosclerosis (60). Recently, OxPLs, which are found to be present in atherosclerotic lesions in humans and mice (61), have also been described to be elevated in circulation and livers of patients and mice with NASH (48). Since it has been demonstrated that only modified LDL and not native LDL has a major influence on the development of atherosclerotic plaques (27), the presence of oxidized lipids and various types of OSEs may represent an essential link between NAFLD and atherosclerosis (34, 62). While systemic dyslipidemia might be responsible for the generation of high levels of OSE and modified LDL, thereby leading to both fatty liver disease and CVD, another possibility is that hepatic lipid accumulation increases oxidative stress and OSEs in the liver, prior to their release into circulation and thus subsequently promoting atherosclerosis development. As lipid peroxidation and consequently the accumulation of altered self-molecules involves interference with structural and functional properties of the physiological state, immunological mechanisms are taking place to protect the body from potential detrimental consequences. In the following section, we provide an overview of existing immune recognition and the pattern recognition receptors (PRRs) responsible for the uptake and/or binding of oxidized lipoproteins and OSEs (63).

## Immune Recognition of Oxidation-Specific Epitopes

Since OSEs identify and label altered proteins and lipids that have been damaged by oxidative stress, cellular debris, and apoptotic cells, recognition mechanisms to provide effective clearance are required (34, 64, 65). Consequently, oxidized lipids and OSEs are recognized by various PRRs on different components of the immune system that mediate their removal to maintain homeostasis in situations of increased oxidative stress. As such, OSEs play an essential role in tissue repair and reconstruction (66). However, during pathological conditions in which OSEs accumulate, they can act as damage-associated molecular patterns (DAMPs), ultimately resulting in chronic inflammation (66). Previous characterization of various OSEs suggested that both cellular and soluble PRRs can recognize OSEs, which we will discuss in light of their involvement in NAFLD and atherosclerosis.

### Cellular Immune Response: Macrophages Orchestrate Inflammation

A variety of cell surface receptors present on innate immune cells recognize OSEs and act as sensors of oxidative stress (67). Here, we will focus on cellular PRRs expressed on macrophages, as they have been shown to play a pivotal role in initiating and sustaining the inflammatory process upon binding and subsequent phagocytosis of oxidatively altered molecules, including oxidized lipoproteins (68, 69).

Toll-like receptors (TLRs) represent a group of classic cellular PRRs of innate immunity capable of binding different pathogen-associated molecular patterns (PAMPs), including bacterial and viral components, as well as DAMPs such as OSEs. For example, oxidized cholesterol esters (OxCE) and OxPL on the surface of extracellular vesicles are ligands for TLR4 (68, 70), while OxPLs have also been reported to stimulate macrophages in a TLR2-dependent mechanism (71). Furthermore, there is evidence that hydro(pero)xylated phospholipids can be considered endogenous TLR4-activating danger signals, and thus TLR4 may act as a sensor for oxidative stress (70). In addition, certain TLRs have been found to respond to OxPL, OxLDL, and other OSEs as part of a multimeric complex with other PRRs (34). As such, it has been shown that the transmission of PC-OxPL-mediated inflammatory signaling requires the formation of a heterotrimer of TLR4-TLR6 and cluster of differentiation 36 (CD36), a scavenger receptor (72, 73). Similarly, ω-(2-Carboxyethyl) pyrrole (CEP) signaling necessitates the cooperation between TLR2 and CD36 (34). Importantly, once ligands bind to TLRs, they activate nuclear factor NF-κB, which stimulates cytokine production and macrophage proliferation (74). Thus, some OSEs represent endogenous ligands recognized by members of the TLR family that can trigger inflammatory responses either with or without cooperation *via* another class of PRRs expressed on macrophages, namely scavenger receptors.

Scavenger receptors comprise another prototypical class of different surface receptors that recognize and internalize OSEs (67). Similar to TLRs, scavenger receptors bind oxidized and non-native LDL particles and contribute to the activation of macrophages in the context of inflammation (67, 75). There are many different types of scavenger receptors, including CD36, scavenger receptor type A1 (SR-A1), SR-A2, SR-B1, CD68, and lectin-like oxidized LDL receptor 1 (LOX1) (67). Of these, SR-A1, SR-A2, and CD36 have shown to be primarily responsible for the uptake of OxLDL, as *in vitro* assays have shown that macrophages deficient in these receptors exhibit 75–90% decreased binding and degradation of OxLDL (76). In addition, PC-OxPs have been found to bind to CD36, whereas PC from non-oxidized phospholipids does not serve as a ligand (77). Moreover, CEP-modified proteins are recognized by CD36 (78) and MDA epitopes are shown to be recognized by SR-A1 and SR-A2 (79, 80).

**Figure 1:**
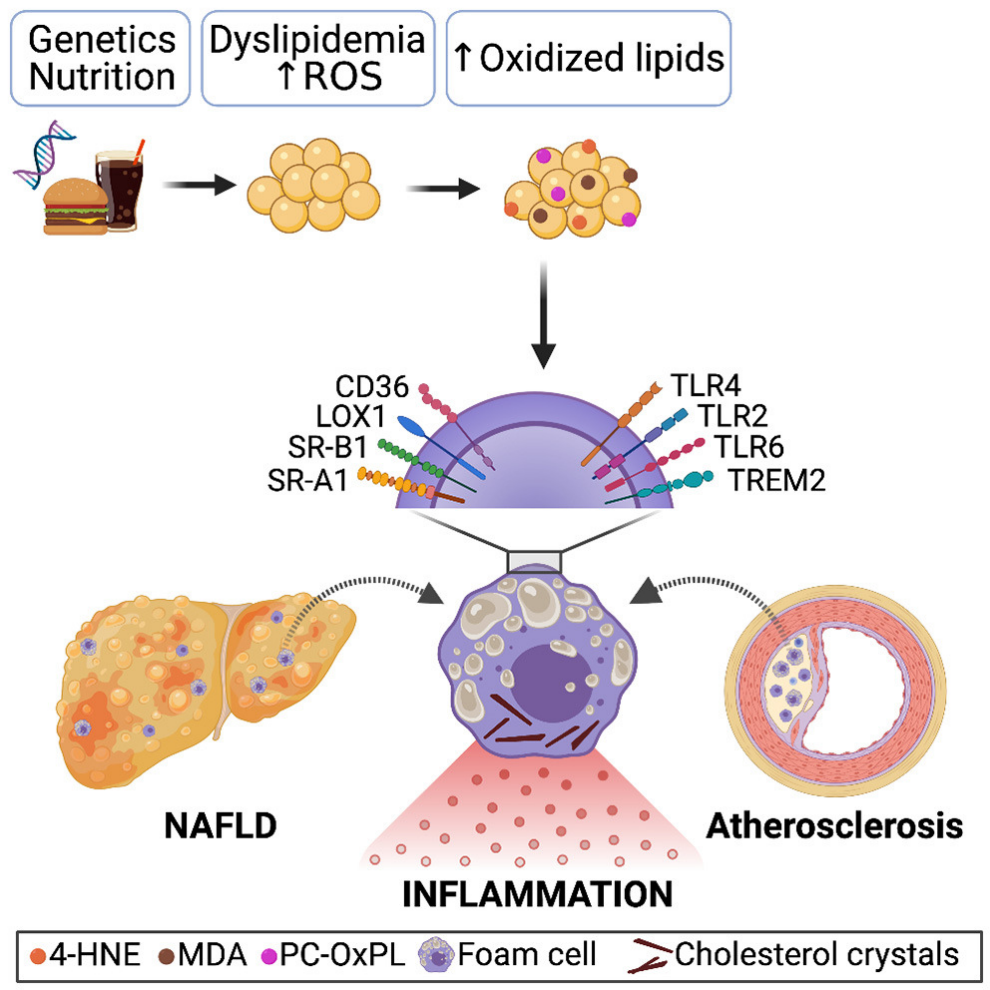
Genetics, sedentary lifestyle and/or unhealthy nutrition causes systemic dyslipidemia characterized by increased LDL levels. Due to oxidative stress and ROS production, oxidation-specific epitopes present on oxidized lipids, dying cells, and microvesicles accumulate. Receptor-mediated recognition and uptake of oxidized lipoproteins by macrophages in the steatotic liver and atherosclerotic plaque results in a foamy appearance, lysosomal dysfunction, and cholesterol crystals formation, causing pro-inflammatory signaling and thereby enhancing disease progression.

Interestingly, receptor-mediated uptake of oxidized lipids by macrophages has been found to play a central role in the chronic inflammatory responses present during both NAFLD and atherosclerosis. In both conditions, excess uptake of oxidized lipoproteins causes a transformation of local macrophages into bloated, lipid-rich foam cells, resulting in the activation of a cascade of pro-inflammatory events (81–84) (Figure 1). Under normal conditions, once internalized, lipids are transported into the lysosomal compartment for degradation by lysosomal enzymes such as cathepsins (85, 86). In contrast to native or acetylated LDL, OxLDL is poorly degraded, leading to disabled intracellular trafficking and lysosomal accumulation of oxidized lipids. Consequently, under pathological conditions characterized by the increased presence of modified lipoproteins and OxLDL, lysosomes turn dysfunctional, and cholesterol crystals are formed, resulting in NLPR3 inflammasome activation, which contributes to inflammation *via* the maturation and release of IL-1β and IL-18 (76, 87, 88). Furthermore, foamy macrophages express more CD36 and SR-A1, the primary receptors mediating OxLDL uptake, and might, in turn, take up even more oxidized lipids, thereby further amplifying inflammation (76). Studies by others and us showed that NLPR3 inflammasome activation contributes to liver disease and atherosclerosis in various murine models, indicating the involvement of this process in both entities (88–91). Moreover, we showed that hematopoietic deficiency of CD36 and SR-A1 reduces foam cell formation and hepatic inflammatory responses during NASH in mice (81, 82). In line, macrophage CD36 and SR-A1/2 contribute significantly to atherosclerotic lesion formation (92, 93). Further, it was demonstrated that *Ldlr*^−/−^ ApoB^100/100^ mice fed a Western diet showed a decrease in atherosclerosis when either SR-A or CD36 was silenced in bone marrow cells using lentivirus vectors encoding shRNA against them (94). Mechanistically, we previously demonstrated that MDA-induced cytokine secretion depends on CD36 and SR-A1. Bone marrow-derived macrophages from mice lacking either of these receptors secrete less CXCL1 upon MDA stimulation, suggesting the involvement of MDA-mediated pro-inflammatory signaling in macrophages in both disease pathologies (46).

In addition to the common findings concerning the contribution of SR-mediated uptake of oxidized lipoproteins during NAFLD and atherosclerosis, multiple studies indicate that TLR-mediated immune recognition also contributes to disease progression. Indeed, NASH has been shown to improve in TLR4 knockout mice receiving a methionine- and choline-deficient (MCD) diet (95), and *Ldlr*^−/−^ mice receiving an atherogenic diet were found protected from triglyceride accumulation in the liver in the absence of TLR4 (96). The contribution of TLR4 to the progression of atherosclerosis is supported by two studies showing that TLR4-deficient *ApoE*^−/−^ mice have lower aortic lipid accumulation (70–80% reduction) and reduced levels of aortic atherosclerosis compared to the control mice (97, 98). In line, a case-control study of 183 patients showed that a single nucleotide polymorphism in *Tlr4* leads to impaired signaling and is associated with reduced plaque formation and a decrease in acute coronary events (99). Moreover, *Tlr4* expression is higher in macrophages in atherosclerotic plaques of *ApoE*^−/−^ mice on an atherogenic diet and in humans (100), suggesting that TLR4 represents a pathophysiological link between oxidized lipids, inflammation, and atherosclerosis. Although most of the available studies focus on the role of TLR4 in NASH and atherosclerosis, it has also been shown that TLR2 deficiency in *Ldlr*^−/−^ mice leads to a reduction in atherogenesis, while administration of TLR2 agonists results in increased atherosclerosis in mice (101). Concerning fatty liver disease, it was demonstrated that administration of an anti-TLR2 antibody ameliorates liver injury, inflammation, steatosis, and fibrosis in rats with obesity (102). Taken together, these studies indicate that TLR signaling contributes to the inflammatory responses observed in NAFLD and atherosclerosis.

More recently, using single-cell RNA sequencing technology, multiple studies in lipid-mediated disorders including NASH and atherosclerosis demonstrate the occurrence of a specialized type of macrophages characterized by high expression of the triggering receptor expressed on myeloid cells 2 (*Trem2*). Whereas, humans and mice with NASH or cirrhosis have increased hepatic expression of *Trem2* that correlates positively with AST and ALT levels (103, 104), more *Trem2*-expressing macrophages are also found in atherosclerotic plaques (105). Interestingly, TREM2 has been shown to bind and recognize (apo-) lipoproteins, including ApoE, LDL, and MDA-LDL particles (106), suggesting TREM2 might also recognize OSEs, thereby potentially contributing to inflammatory responses and disease progression. Functional studies assessing the role of TREM2 in NAFLD and atherosclerosis are still required since this receptor might also be involved in a common lipid-induced mechanistic pathway.

Taken together, there are overarching mechanisms regulating the uptake of OSEs and OxLDL *via* PRRs on macrophages. As a result, macrophages and hepatic Kupffer cells turn into foam cells, become activated, and secrete pro-inflammatory factors in response to elevated oxidized lipids, thereby contributing to the development of NAFLD and atherosclerosis in a similar process, representing part of a shared etiology. In the next section, we will focus on soluble factors recognizing OSEs and their involvement in steatohepatitis and atherosclerosis.

### Humoral Immune Response: B Cells Enter the Stage

Besides recognizing OSEs by cell surface receptors, soluble receptors capable of binding OSEs are described, such as C-reactive protein (CRP) and proteins of the complement system (62, 107). CRP, an acute-phase protein produced in the liver, was found to recognize PC-OxPL on OxLDL and apoptotic cells, indicating CRP's responsiveness to OSEs (108). Since CRP levels are a known marker of systemic inflammation, CRP in circulation might indirectly also be considered a reflection of tissue injury, oxidative stress, and/or ongoing lipid peroxidation and OSE levels. As a regulator of complement activity, an essential machinery for clearance of metabolic waste and dead cells, complement factor H (CFH) was identified to recognize and bind MDA epitopes. Moreover, genetic variants of one of the MDA-binding sites of CFH were shown to influence the capacity of CFH to bind MDA (109). Since MDA has pro-inflammatory effects during NASH, reduced CFH might contribute to the harmful effects of lipid peroxidation products in fatty liver disease progression. Nevertheless, the functional role of CFH-mediated OSE recognition and its consequences for NAFLD and CVD has not yet been fully described.

Insights from others and us give prominence to B cell-derived antibodies or immunoglobulins targeting OSEs in NAFLD and CVD (59, 107, 110–112). Antibodies exist in different isotypes (IgM, IgG, IgA, IgE, IgD) that implement key functions in defending the body against pathogens and are also responsible for maintaining homeostasis by eliminating metabolic waste (113). Owing to their broad specificity for pathogens and their ability to recognize highly conserved structures such as self-antigens, they provide another group of soluble factors able to detect OSEs residing on oxidized lipoproteins, apoptotic cells, and extracellular vesicles (64, 114). Studies in mice and humans have shown that various OSEs, including PC-OxPL, MDA, and 4-HNE adducts, are prominent targets of natural antibodies, which are pre-existing germline-encoded antibodies, predominantly of the IgM type, that are present without external antigens. In fact, several OSEs are bound by up to 30% of all natural IgM found in the plasma of both wild-type and gnotobiotic mice (64, 114), indicating their relevance for homeostasis and immune defense. In addition to innate natural IgM antibodies, produced and secreted by B1 cells, adaptive IgG isotypes secreted by B-2 cells are capable of recognizing and binding various OSEs such as MDA (115).

Interestingly, consistent observations concerning systemic levels of antibodies binding OxLDL and OSE are described between NAFLD and atherosclerosis. First of all, in line with the negative association between IgM levels in circulation and the severity of atherosclerosis and CVD in general (107), we have shown that NAFLD patients have lower serum IgM titers toward various OSEs including MDA and MAA compared to healthy controls (59). While liver disease data upon IgM deficiency is still lacking, a lack of secreted IgM antibodies resulted in increased atherosclerotic plaques in *Ldlr*^−/−^ mice after Western-type diet (112). In addition, we previously demonstrated that an increase in B1-derived natural IgM with specificity for OxLDL in *Ldlr*^−/−^ mice deficient for sialic acid-binding immunoglobulin-like lectin G (Siglec-G) protects against atherosclerosis and steatohepatitis after atherogenic diet, further supporting the protective role of IgM antibodies (110). By recognizing and neutralizing OSEs, IgM can limit OxLDL-induced foam cell formation, as well as pro-inflammatory macrophage responses, and generally contribute to reduced inflammation (115). More studies pointing out the protective properties of increased anti-OxLDL IgM levels are described in the section about immunotherapy.

Besides altered IgM antibody levels, ~40% of adults (49) and 60% of children have elevated IgG titers in circulation when diagnosed with NAFLD (116). Particularly, anti-OSE IgGs produced by B-2 lymphocytes have been shown to correlate with the onset of steatohepatitis and are considered as an independent predictor of fibrosis in NAFLD patients (49, 116, 117). These observations are in line with those in atherosclerosis, where higher IgG levels are associated with more severe atherosclerosis in humans and mice (118–120). Furthermore, a meta-analysis even suggests that systemic IgG antibody titers are a potential predictor of future atherosclerosis-related cardiovascular events since a positive correlation between IgG levels and events was found (121). However, the functional significance and contribution of IgG are still unclear as studies in humans and animal models indicate that anti-OSE IgG may also have atheroprotective properties (107, 122, 123). Given these diverse and partly inconsistent findings, the role of IgG in atherosclerosis has not been fully elucidated, which has been discussed in detail by Sage et al. (124). Nevertheless, in mice, anti-CD20 antibody-mediated depletion of B cells reduces the development of atherosclerosis (125), and ameliorates NASH progression (126). Importantly, anti-CD20 treatment preserves the production of anti-OxLDL IgM antibodies, while IgG targeting OxLDL are greatly diminished (125). In addition, TACI-Ig mice, characterized by a depletion of B2 cells, were found to have milder steatohepatitis and less progression of fibrosis (117), further supporting a more pro-inflammatory effect of anti-OSE IgG antibodies.

To summarize, altered anti-OSE IgM and IgG titers are associated with NAFLD and fibrotic NASH, consistent with findings in atherosclerosis (50, 59, 107, 127), suggesting another pathophysiological pathway in common between NAFLD and atherosclerotic CVD. While IgG-producing B-2 cells may promote pro-inflammatory mechanisms (50), natural IgM secreted by B1 cells seems to have protective properties (50). As such, antibodies recognizing OSEs provide an attractive target for B cell-mediated immunotherapeutic approaches to improve therapeutic strategies and/or prevent cardiovascular complications during NAFLD. Besides targeting B cells as done using anti-CD20 administration, immunization approaches to enhance immunity against OSEs have been investigated, which we will summarize in the next section.

## Targeting Oxidized Lipids as Immunotherapeutic Approach

**Figure 2:**
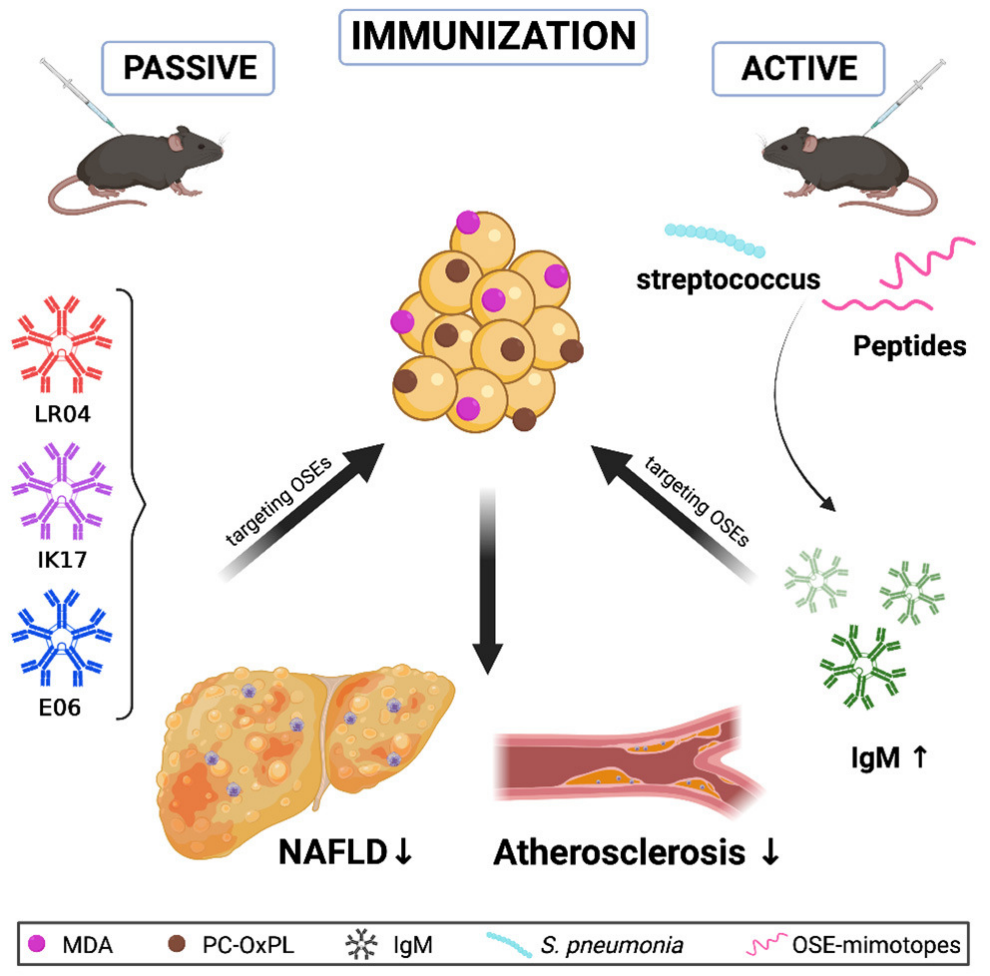
In mice, passive immunization strategies with OSE-recognizing IgM antibodies LR04, IK17, and E06, or boosting IgM antibody titers toward oxidized lipids in active immunization approaches, such as with heat-inactivated *S. pneumococci* or specific peptides, can induce protection against NAFLD progression and reduce atherosclerotic plaque burden.

As neither lifestyle modification nor currently existing pharmacotherapy is sufficient to reduce liver fibrosis and inflammation, NAFLD is becoming a global burden on the healthcare system and poses an urgent need for developing therapeutic interventions (128). Although several drugs and combination therapies are under investigation, no truly effective treatment has yet been identified (129, 130). However, regarding oxidative stress, nutrients and antioxidants such as vitamin E have beneficial effects on NAFLD (131) by lowering the NAFLD activity score (NAS) and reducing inflammation (132, 133). Consistent with these findings, a recent study has demonstrated that vitamin E is negatively correlated with serum MDA in women suffering from NAFLD (54). In addition, similar outcomes also indicate a protective role of vitamin E in atherosclerosis (134, 135). Nevertheless, since we currently lack complete understanding of the metabolic pathways affected and controlled by oxidative stress, more studies are needed to design an adequate therapeutic trial to assess antioxidants to combat atherogenesis (136, 137). Growing evidence shows that directly addressing OSEs yields a high potential for reducing the inflammatory response in NAFLD and atherosclerosis, thereby representing a promising avenue for treating both diseases (48, 63, 138). Here, we will provide an overview of current data on intervention approaches addressing OSEs through immunization strategies. Simplified, enhancing protective B cell antibody responses *via* immunization can be divided into two separate classes: passive immunization in which antibodies that directly target, bind, and inactivate an antigen are infused; and active immunization, wherein an antigen is used in a vaccination protocol to boost antibody titers and as such, provide long-term immunity (Figure 2).

### Passive Immunization

As described above, data from others and us support that anti-OSE IgM antibodies act protective against liver disease and atherosclerosis, and as such, are a viable tool to prevent disease (59, 83, 110, 127, 139). Besides our findings using *Siglec-G*^−/−^ mice that have increased anti-OSE IgM levels, we showed that intravenous administration of LR04, a monoclonal IgM specifically targeting MDA, neutralizes endogenously produced MDA epitopes, leading to a decrease in liver inflammation in *Ldlr*^−/−^ mice after a Western-type diet (46). Similarly, intravenous injection and intraperitoneal infusion of the human antibody IK17, directed to MDA-like epitopes, significantly reduced atherosclerosis progression by 30–50% (140, 141). Moreover, antibodies E06 and T15, which target PC epitopes on OxPL were found to protect against atherosclerosis by blocking lipid uptake by macrophages, preventing inflammation, and promoting the clearance of apoptotic cells (141). Expression of the single-chain variable fragment of E06 in *Ldlr*^−/−^ mice was shown to be sufficient to suppress the development of atherosclerosis at multiple disease stages (139). These mice were also protected against various aspects of NASH, including steatosis, inflammation, fibrosis, hepatocyte cell death, and progression to hepatocellular carcinoma, further supporting the causal role of OxPL in the pathogenesis of NASH (49).

Besides evaluating the potential beneficial effect of IgM antibodies on disease outcome, certain studies focused on using IgG antibodies. For example, administration of the human IgG antibody 2D03 directed against MDA-modified ApoB exhibited atheroprotective immune responses against OxLDL (123). More recently, a study revealed the protective function against the development of atherosclerotic plaques using autoantibodies against the ApoB100 peptide p210 in *ApoE*^−/−^ mice, which was accomplished by injecting IgG2b against p210. These results in *ApoE*^−/−^ mice support previous human studies, which showed an inverse association between apoB100 native p210 IgG and plaques in coronary or carotid arteries (142).

### Active Immunization

Besides passive immunization approaches, several studies assessed the potential beneficial effect of enhanced immunity toward various epitopes of OxLDL using these as antigens. In one of the first studies applying active immunization, injection of homologous MDA-LDL into *Ldlr*^−/−^ rabbits induced higher anti-MDA antibody titers and significantly reduced the extent of atherosclerotic lesions in the aortic tree of immunized animals (143). Consistent with this, *Ldlr*^−/−^ mice injected with MDA-modified LDL showed smaller atherosclerotic lesion size after an atherogenic diet, although this was independent of changes in anti-OSE IgM levels (144). In relation to fatty liver disease, a study in which C57BL/6 mice were injected with MDA conjugated to bovine serum albumin (MDA-BSA) reported increased severity of NASH in immunized mice after MCD diet. The authors attributed this to the fact that MDA-BSA injection enhanced IgG responses and increased hepatic T cell infiltration, which may ultimately lead to increased inflammation (145). Importantly, immunization with MDA-BSA adducts did not influence IgM antibody levels toward MDA-derived antigens, potentially lacking protective capacities. On the other hand, studies that immunized *Ldlr*^−/−^ mice with heat-inactivated *S. pneumonia*, which significantly induced PC-OxPL recognizing IgM titers due to molecular mimicry, showed that mice immunized were protected against diet-induced steatohepatitis and atherosclerosis after Western-type diet (83, 146), supporting the idea that raising IgM titers toward OxLDL has beneficial effects.

In addition to using various OSEs as antigens, several experimental studies assessed whether administration of stable peptides could be applied for immunization. One of these peptides is the p210-PADRE, which has already been mentioned in the section on passive immunization. Besides direct infusion of IgG antibodies, ApoE^−/−^ mice were immunized with the p210-PADRE peptide, which induced a specific IgG1 response against p210, thereby preventing MDA-LDL accumulation in lesions and reducing atherosclerotic plaque formation in the aorta (142). Moreover, immunization with OxLDL- and MDA-modified ApoB100 peptides were described to have an atheroprotective effect associated with an increase in IgG and IgM antibodies specific for the antigen used (142, 147). Interestingly, two small immunogenic peptides, linear P1 and circular P2, were identified that immunologically mimic MDA-type epitopes (148). Since we have shown the critical role for endogenous MDA in NASH and P2-BSA immunization raised IgM antibody levels toward MDA, these mimotopes are a promising tool to induce immunity against this relevant antigen to reduce/prevent NAFLD progression (46). Although most of the above-mentioned immunization approaches have been reported to be successful for atherosclerosis, more studies focusing on liver disease are required to further confirm that the treatment principles of increased anti-OSE IgM to reduce atherosclerosis also apply to NASH.

## Outlook

Growing evidence indicates a significant association between the clinical patterns of NAFLD and atherosclerosis. Common molecular mechanistic pathways seem to play a central role in disease progression, and NAFLD might even be considered a risk factor for developing CVD.

Just as receptor-mediated uptake of oxidized lipids leads to macrophages' foamy appearance and pro-inflammatory processes is one consistent observation in the liver during NAFLD and the vessel wall during atherosclerosis, other mechanistic patterns might be similarly occurring in both pathologies (81–84). Besides, in both NAFLD and atherosclerosis, B-1-derived IgM antibodies seem to have a protective role, while IgG produced by B2 cells seems to promote inflammation (115). Therefore, studies assessing both conditions might significantly enhance our understanding of their interrelationship and potentially lead to the identification of novel targets to improve treatment strategies. In particular, the *Ldlr*^−/−^ mice on a high-fat, high-cholesterol diet is a suitable model to study the underlying pathways occurring in fatty liver disease and linked atherosclerosis (149). We have previously shown that these mice develop steatotic livers with increased inflammation and oxidative stress already after 2 weeks of diet, while atherosclerotic plaques develop after 6–8 weeks of dietary intervention (46). Moreover, *Ldlr*^−/−^ mice present a human-like lipid profile, which is essential to facilitate translational studies regarding lipid-mediated diseases (150). In addition, since CVD is still the primary cause of death in patients with early NAFLD (9, 10), assessing liver disease in human cardiovascular study cohorts and vice versa could further enhance our understanding and potentially identify patients with advanced disease stage and/or increased risk for cardiovascular events. In this review, we focused on the potential immunotherapeutic approach by enhancing immunity toward OSEs and oxidized lipids. Since similar observations for systemic anti-OSE antibody titers are described, one might hypothesize that results from immunization studies, which mostly come from work in the field of atherosclerosis, can be translated and tested to prevent NAFLD progression. Nevertheless, further studies are needed to understand the role of OSEs and OSE-reactive immunity in maintaining homeostasis and controlling inflammatory responses to design translational studies and eventually offer novel treatment strategies for patients.

## Author Contributions

CH and TH wrote and edited the manuscript. TH composed the figures. DR helped with figure composition. All authors contributed to the article and approved the submitted version.

## Funding

TH was funded by a Veni (NWO; 91619012), MLDS Right-On-Time grant (MLDS; 19-28), and Zukunftskollegs grant (FWF; ZK81B).

## Conflict of Interest

The authors declare that the research was conducted in the absence of any commercial or financial relationships that could be construed as a potential conflict of interest.

## Publisher's Note

All claims expressed in this article are solely those of the authors and do not necessarily represent those of their affiliated organizations, or those of the publisher, the editors and the reviewers. Any product that may be evaluated in this article, or claim that may be made by its manufacturer, is not guaranteed or endorsed by the publisher.

## Abbreviations

4-HNE: 4-Hydroxynonenal
BSA: Bovine serum albumin
CD36: Cluster of differentiation 36
CEP: ω-(2-Carboxyethyl) pyrrole
CFH: Complement factor H
CRP: C-reactive protein
CVD: Cardiovascular disease
DAMP: Damage-associated molecular pattern
FFA: Free fatty acid
HDL: High-density lipoprotein
IgG: Immunoglobulin type G
IgM: Immunoglobulin type M
IL: Interleukin
LDL: Low-density lipoprotein
LDLR: Low-density lipoprotein receptor
MAA: Malondialdehyde-acetaldehyde
MDA: Malondialdehyde
NADPH: Nicotinamide adenine dinucleotide phosphate
NAFLD: Non-alcoholic fatty liver disease
NAS: NAFLD activity score
NASH: Non-alcoholic steatohepatitis
OSE: Oxidation-specific epitope
OxLDL: Oxidized LDL
OxPL: Oxidized phospholipids
PAMP: Pathogen-associated molecular pattern
PC-OxPL: Phosphocholine-containing OxPL
ROS: Reactive oxygen species
SR: Scavenger receptor
TBAR: 2-Thiobarbituric acid reaction
TLR: Toll-like receptor
TREM2: Triggering receptor expressed on myeloid cells 2
VLDL: Very low-density lipoprotein.
